# Altered Intracellular Localization of SOD1 in Leukocytes from Patients with Sporadic Amyotrophic Lateral Sclerosis

**DOI:** 10.1371/journal.pone.0075916

**Published:** 2013-10-14

**Authors:** Cristina Cereda, Emanuela Leoni, Pamela Milani, Orietta Pansarasa, Giuliano Mazzini, Stefania Guareschi, Elena Alvisi, Andrea Ghiroldi, Luca Diamanti, Stefano Bernuzzi, Mauro Ceroni, Emanuela Cova

**Affiliations:** 1 Laboratory of Experimental Neurobiology, “C. Mondino” National Neurological Institute, Pavia, Italy; 2 Department of Brain and Behavioral Science, University of Pavia, Pavia, Italy; 3 IGM-CNR, Histochemistry and Cytometry, Department of Animal Biology, University of Pavia, Pavia, Italy; 4 Division of General Neurology, “C. Mondino” National Neurological Institute, Pavia, Italy; 5 Immunohematological and Transfusional Service and Centre of Transplantation Immunology, IRCCS Foundation “San Matteo”, Pavia, Italy; Inserm, France

## Abstract

Several lines of evidence support the hypothesis of a toxic role played by wild type SOD1 (WT-SOD1) in the pathogenesis of sporadic amyotrophic lateral sclerosis (SALS). In this study we investigated both distribution and expression profile of WT-SOD1 in leukocytes from 19 SALS patients and 17 healthy individuals. Immunofluorescence experiments by confocal microscopy showed that SOD1 accumulates in the nuclear compartment in a group of SALS subjects. These results were also confirmed by western blot carried out on soluble nuclear and cytoplasmic fractions, with increased nuclear SOD1 level (*p*<0.05). In addition, we observed the presence of cytoplasmic SOD1 aggregates in agreement with an increased amount of the protein recovered by the insoluble fraction. A further confirmation of the overall increased level of SOD1 has been obtained from single cells analysis using flow cytometry as cells from SALS patients showed an higher SOD1 protein content (*p*<0.05). These findings add further evidence to the hypothesis of an altered WT-SOD1 expression profile in peripheral blood mononuclear cells (PBMCs) from patients with ALS suggesting that WT-SOD1 species with different degrees of solubility could be involved in the pathogenesis of the disease.

## Introduction

Amyotrophic lateral sclerosis is a rare adult-onset neurodegenerative disease characterized by degeneration of motor neurons in the motor cortex, brainstem and spinal cord. As other neurodegenerative diseases, in the majority of cases there is no apparent genetic linkage (a form of the disease referred to as sporadic ALS, SALS), although mutations in some causative genes have been also found in SALS [1]. SOD1 remains one of the distinctive genes for familial ALS (FALS) since the other genes (*FUS/TLS*, *TARDBP*, *C9ORF72* etc.) [2]–[12] are also associated with other diseases, especially with frontotemporal dementia [13]–[16].

Although both familial and sporadic ALS patients show some degree of heterogeneity as far as symptoms, age of onset, and disease duration, FALS are indistinguishable from SALS cases on the basis of clinical and instrumental criteria. FALS and SALS also present a remarkably similar set of pathological hallmarks at cellular level and some findings reported the existence of shared pathogenic mechanisms involving SOD1 protein. Specifically, misfolded SOD1 was observed in both FALS and SALS patients hinting to the hypothesis that conformational modification of wild-type SOD1 (WT-SOD1) could be involved in the sporadic form of the disease similarly to mutant SOD1 (mut-SOD1) in the familial cases [17].

The hypothesis that altered WT-SOD1 may be implicated in SALS pathogenesis was proposed for the first time after the discovery of a 32 kDa endogenously modified SOD1 species in the spinal cord extracts of SALS patients [18]. A positive reaction to antibodies raised against a mut-SOD1 conformational epitope or denatured SOD1 was also found in motor neurons from SALS patients expressing WT-SOD1 [19], [20]. Others [21] described a misfolded SOD1 in the form of granular aggregates in nuclei of glial cells from SALS cases while a iper-oxidized misfolded SOD1 was found aggregated with the anti-apoptotic protein Bcl-2 in the cytoplasm of lymphoblasts from sporadic ALS patients. The same aberrant link has been previously described between mut-SOD1 and Bcl-2 [22] thus adding further evidence of a pathogenic mechanism common to both WT- and mut-SOD1. Notably, following environmental insults, such as oxidative stress, WT-SOD1 may change its physiological conformation gaining some of the toxic functions exerted by mut-SOD1 [23]. However, under stressful conditions, WT-SOD1 is more efficient in protecting against cellular damage compared to mut-SOD1 [24].

In previous works [25], [26] we also obtained evidence for an involvement of WT-SOD1 in sporadic cases since a reduced SOD1 expression profile in lysates from peripheral blood mononuclear cells (PBMCs) was observed. Nevertheless, abnormally high levels of SOD1 transcript were found in the same cells of sporadic patients thus raising the question about the discordance between protein and mRNA expression levels [25]. To explain this discrepancy, we hypothesized a re-localization of the “missing” protein in other cellular compartments, such as the nucleus, or, alternatively, its precipitation in the insoluble fraction not detectable with our previous isolation protocol since low-strength detergent buffers were used in those experiments.

Here we aim to clarify the observed discrepancy between SOD1 protein and mRNA level by studying its localization and aggregation in different compartments of PBMCs from SALS patients. The evidence that peripheral tissues display signs of central nervous system diseases, such as ALS and other neurodegenerative pathologies [27]–[32] strongly sustains their use. The final aim of our study was to investigate SOD1 subcellular distribution and appearance in PBMCs from sporadic cases of ALS. Our study could provide further evidence about WT-SOD1 peripheral signature in the most common form of ALS, opening new perspectives on the stratification of patients in homogeneous subgroups.

## Materials and Methods

### Subjects

The study protocol was approved by the Ethical Committee of the IRCCS, National Neurological Institute “C. Mondino” of Pavia (Italy); before being enrolled, subjects participating in the study signed an informed consent form. Experiments were done using PBMCs isolated from 19 SALS patients (mean age: 58.2±13). ALS diagnosis was made according to the revised El Escorial Criteria [33] at the IRCCS National Neurological Institute “C. Mondino”. SALS individuals harboring mutations in the *SOD1*, *FUS/TLS*, *TARDBP*, *C9ORF72* and *ANG* genes were excluded from this study. Patients' characteristics are reported in Table 1.

**Table 1:** Demographic and clinical data of patients with ALS.

Case number SALS#	Sex	Age at onset (years)	Disease duration (months)	ALSFRS-R score (at blood withdrawal)	Site of onset	Comments
1	M	53	18	30	S	
2	F	54	36	38	S	
3	F	55	15	21	S	
4	F	45	30	42	S	
5	F	62	23	28	B	
6	M	56	17	28	B	
7	M	77	13	13	S	
8	F	59	32	32	B	
9	M	73	17	35	B	deceased
10	F	62	8	32	B	
11	M	67	8	28	B	
12	F	57	30	40	S	
13	M	79	10	32	S	
14	M	64	57	17	B	deceased
15	M	72	104	30	S	
16	M	55	46	28	S	
17	M	27	153	38	S	
18	M	38	62	32	S	
19	M	67	27	17	B	

*Legend*: M: male, F: female, S: spinal, B: bulbar.

Seventeen sex- and age-matched healthy volunteers free from any pharmacological treatment (mean age: 50.5±13.1) were recruited at the Immunohematological and Transfusional Service and Centre of Transplantation Immunology IRCCS Foundation “San Matteo” (Pavia, Italy) and used as non-neurological controls (CTR). PBMCs were also collected from 7 patients affected by a different neurodegenerative disease, i.e. Alzheimer's disease (AD, mean age: 71.4±8.8), and used as neurological controls to assay disease specificity of the evaluated parameters. Diagnosis of AD was based on criteria expressed by Aging-Alzheimer's Association workgroups [34]. All the subjects were assayed to rule out the presence of inflammatory diseases by white blood cell counts, where subjects with WBCs >11×10^9^ were excluded from the study.

### Isolation of PBMCs

Peripheral blood mononuclear cells were immediately isolated from peripheral venous blood by Histopaque®-1077 (Sigma-Aldrich, Italy) following manufacturer's instructions. PBMCs (composed of ∼80% lymphocytes and ∼20% of monocytes) were washed twice in phosphate buffer saline solution (PBS) and centrifuged at 1200× g for 8 min at room temperature. Cells viability was assessed by trypan blue exclusion test. Aliquotes of PBMCs were recovered from each subject and processed for confocal immunofluorescence, subcellular fractionation and flow cytometry experiments (1×10^5^, 10×10^6^ and 5×10^5^ cells, respectively). Cases and controls were collected in the same conditions and at similar timing in order to avoid or minimize any bias. Furthermore, patients and controls were assayed to rule out the presence of inflammatory diseases, as previously reported [35].

### Immunofluorescence analysis of SOD1 in PBMCs

Freshly isolated PBMCs were harvested, washed with PBS and suspended in RPMI-1640 medium. About 1×10^5^ cells were placed on pre-coated poly-L-Lysine slide (Thermo Fisher Scientific Inc., MA, USA) and incubated at 37°C for 15 min to allow them to attach to the slide. Cells were rinsed twice with PBS and then fixed using a solution of 4% paraformaldehyde in PBS for 15 min at room temperature (RT), as previously published [36]. Fixed cells were washed with PBS three times for 5 min. Samples were then treated with a blocking solution containing 5% goat serum in 0.1% Tween-PBS for 1 hr to block unspecific protein binding sites and were incubated with the primary antibody (rabbit polyclonal anti-SOD1, FL-154 from Santa Cruz Biotechnology, CA, USA; dilution 1∶250 in blocking solution) overnight (o/n) at 4°C. Cells were then washed with PBS, three times for 5 min, and incubated with secondary antibody (Alexa 488 goat anti-rabbit, Invitrogen, Carlsbad, CA, USA; dilution 1∶700 in blocking solution), 1 hr at RT. Samples were finally washed with PBS (three times for 5 min) and incubated for 30 s with 4′,6′ diamino-2-phenylindole (DAPI) solution (Sigma-Aldrich, Italy; dilution 1∶2500 in deionized water). After being washed twice, slides were mounted with 60% glycerol solution, dried for 2 hrs and nail-polished. Localization of SOD1 protein was defined using confocal laser microscope using z-stack acquisition (Leica DM IRBE, Leica Microsystems Srl, Italy).

### Subcellular fractionation and protein extraction

To separate the soluble cytoplasmic fraction (SCF) and the soluble nuclear fraction (SNF) of the cellular proteins, pellets were lysed as previously described [37]. After the separation of the two soluble fractions, the remaining pellet representing the insoluble cellular fraction (ICF) was resuspended in lysis buffer (50 mM Tris HCl pH 7.4, 150 mM NaCl, 1% Triton X-100, 1% protease inhibitors, 40 mM NaF and 1 mM Na_3_VO_4_), as described by [38]. Before freezing at −80°C, protein content was determined by Bradford method (Sigma-Aldrich, Italy) and the working fractions were aliquoted.

### Determination of SOD1 expression by western blot

Western blot analysis was performed by SDS-polyacrylamide gel electrophoresis (PAGE) by loading 25 µg of total proteins into a 12.5% SDS-PAGE gel. Fractioned proteins were transferred to a nitrocellulose membrane (Trans-blot, BioRad Laboratories, Italy) using a liquid transfer apparatus (BioRad) and blocked with 5% non-fat dry milk in Tween-20 Tris-Buffered Saline solution (TBS-T) for 1 hr. The membranes were incubated overnight with a rabbit polyclonal primary antibody anti-SOD1 (Santa Cruz Biotechnology, Inc., USA; dilution 1∶1000) in blocking solution. Immunoreactivity was detected using donkey anti-rabbit secondary peroxidase-conjugated antibody (GE Healthcare, UK; dilution 1∶10000) and bands were visualized using enhanced chemiluminescence detection kit (ECL Advance, GeHealthcare, UK). For subsequent immunoreaction, primary and secondary antibodies were removed from the membrane following an incubation for 20 min in stripping solution (mercaptoethanol, 2% SDS, and 62.5 mM Tris/HCl, pH 6.7), washing with TBS-T (3 times for 10 min), and then the membranes were processed as described above. Equal amount of proteins from SCF, SNF and ICF were verified by reprobing the membranes with antibodies against lactate dehydrogenase (LDH, Santa Cruz Biotechnology, Inc., USA; dilution 1∶1000), proliferating cell nuclear antigen (PCNA, Calbiochem, USA; dilution 1∶1000), ,and β-tubulin (β-TUB, Santa Cruz Biotechnology, Inc., USA; dilution 1∶250), respectively. Membranes were also tested to exclude cross-contamination between cytoplasmic and nuclear fractions.

Densitometric analysis of the bands was performed using Quantity One software (BioRad, Italy). After background subtraction, intensity values corresponding to each band have been generated. Densitometric assessment in arbitrary units (ratio of the value of SOD1 with the correspondent value of the loading control i.e. LDH, PCNA, and β-tubulin for cytosolic, nuclear and insoluble fractions, respectively) has been carried out in each cellular fraction.

### Determination of SOD1 expression by flow cytometry

A set of two tubes, each one containing 5×10^5^ freshly isolated PBMCs, was treated with 4% paraformaldehyde solution for 15 min at RT in agitation. After washing twice with PBS, cells were suspended in blocking buffer (1% BSA in 0.1% Triton X-100 PBS) and kept for 30 min at RT with gentle agitation. Primary anti-SOD1 antibody (rabbit polyclonal anti-SOD1, FL-154, Santa Cruz Biotechnology, CA, USA; dilution 1∶250 in blocking solution) was added and PBMCs incubated o/n at 4°C. After centrifugation and washing twice with PBS, secondary antibody (Alexa 488 goat anti-rabbit Invitrogen, CA, USA; dilution 1∶700 in blocking solution) was left for 1 hr at RT and then cells were rinsed and finally suspended in PBS. Control tubes were processed at the same time and treated likewise, but in the absence of the primary antibody. Measurements were done with a PartecPAS II flow cytometer (Munster, Germany). A laser 488 nm blue line was used to excite Alexa for SOD1 analysis. A preliminary instrument alignment and control was always set up (with rat thymocytes stained with Propidium Iodide) to ensure the best instrumental analytical performance. Immediately before measurement, each sample was filtered through Filcons 100 (ConsulTS, Turin, Italy) to remove cell clusters. For a sample measurement, a minimum of 20,000 events were acquired. Data were displayed as a fluorescence histogram of the green fluorescence FL1 signals (SOD1). A digital converter allowed data transfer to the PC installed into the flow cytometer whose specific software Flow Max was dedicated to the quantitative determination of interested target cells.

### Selection of a cut-off value

After analysing the distribution of the values obtained from western blotting corresponding to the nuclear SOD1 we observed a bell-shape distribution in CTR (Figure S1) whereas a bimodal distribution was obtained in ALS. Being the mean value of the curve with the normal distribution (mean = 3.1) at the highest frequency point between the two distribution peaks, we ascertained to use this value as arbitrary cut-off. A similar approach was already published in the literature [39].

### Statistical analysis

Statistical differences between SALS, neurological and healthy controls were evaluated using ANOVA analysis. Comparisons between two groups (SALS and healthy controls) were made by applying Student's t-test for unpaired data. Mann-Whitney test was also carried out for a further sensitivity analysis. Standard regression analysis was used to determine the relationship between patients' clinical features and nuclear SOD1 expression. All statistical analyses were carried out with Graph Prism 5.0 statistical program. A *p* value<0.05 was considered statistically significant.

## Results

### SOD1 localization in PBMCs by immunofluorescence

**Figure 1:**
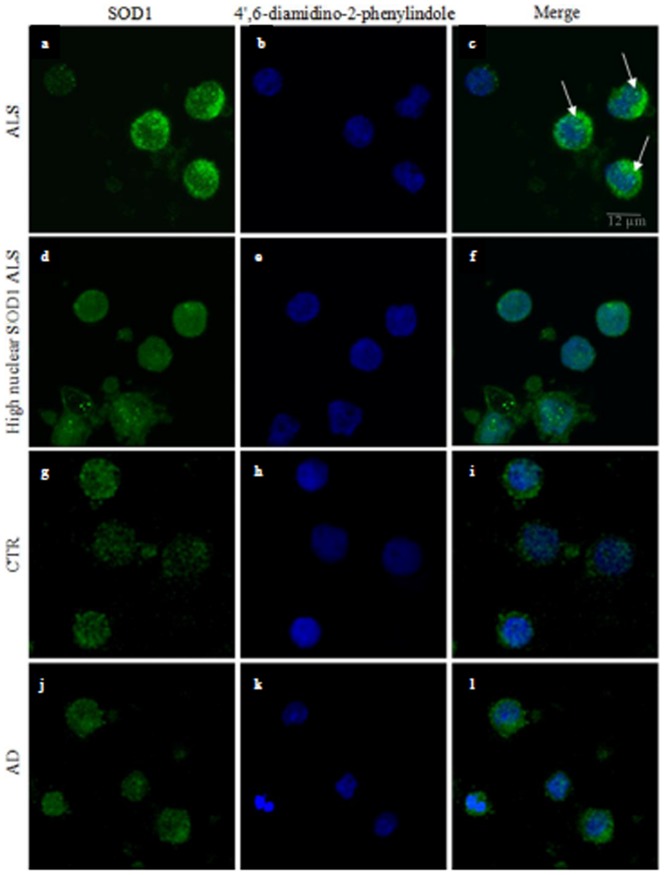
Confocal images of SOD1 distribution in PBMCs from ALS, CTR and AD subjects. Immunofluorescence images reveal SOD1 cytoplasmic aggregates in SALS patients with lower SOD1 nuclear distribution (panels a–c) while other patients showed a higher SOD1 signal in the nuclear compartment (panels d–f). The distribution of SOD1 in PBMCs from CTR (panels g–i) was homogeneous in both the cellular compartments and a lower fluorescence intensity was also observed. A diffuse pattern of SOD1 distribution in nucleus and cytoplasm is clearly shown in AD patients (panels j–l). Original magnification: ×600.

In previous works we found that PBMCs from SALS patients expressed reduced soluble SOD1 level even if a significant increase of mRNA amount was observed [25], [26], [29]. This discrepancy prompted us to investigate SOD1 sub-cellular distribution and appearance by a morphological technique, such as confocal microscopy. Immunofluorescence of PBMCs followed by confocal analysis was performed in SALS patients, CTR and AD patients to morphologically evaluate nuclear and cytoplasmic localization of SOD1. The presence of brighter SOD1 signal in ALS PBMCs compared to both neurological and healthy control cells was evident (Figures 1a, d, g, l). Moreover, the presence of SOD1 aggregates, mostly perinuclear, was observed in the cytoplasm of the ALS group with lower SOD1 nuclear signal (Figures 1a, b, c). Additionally, we identified a group of twelve ALS patients characterized by a higher nuclear SOD1 signal (Figures 1d, e, f and Figure S2). We did not witness any change in nuclear size between ALS cases and controls.

### Nuclear and cytoplasmic soluble SOD1 expression in PBMCs

**Figure 2:**
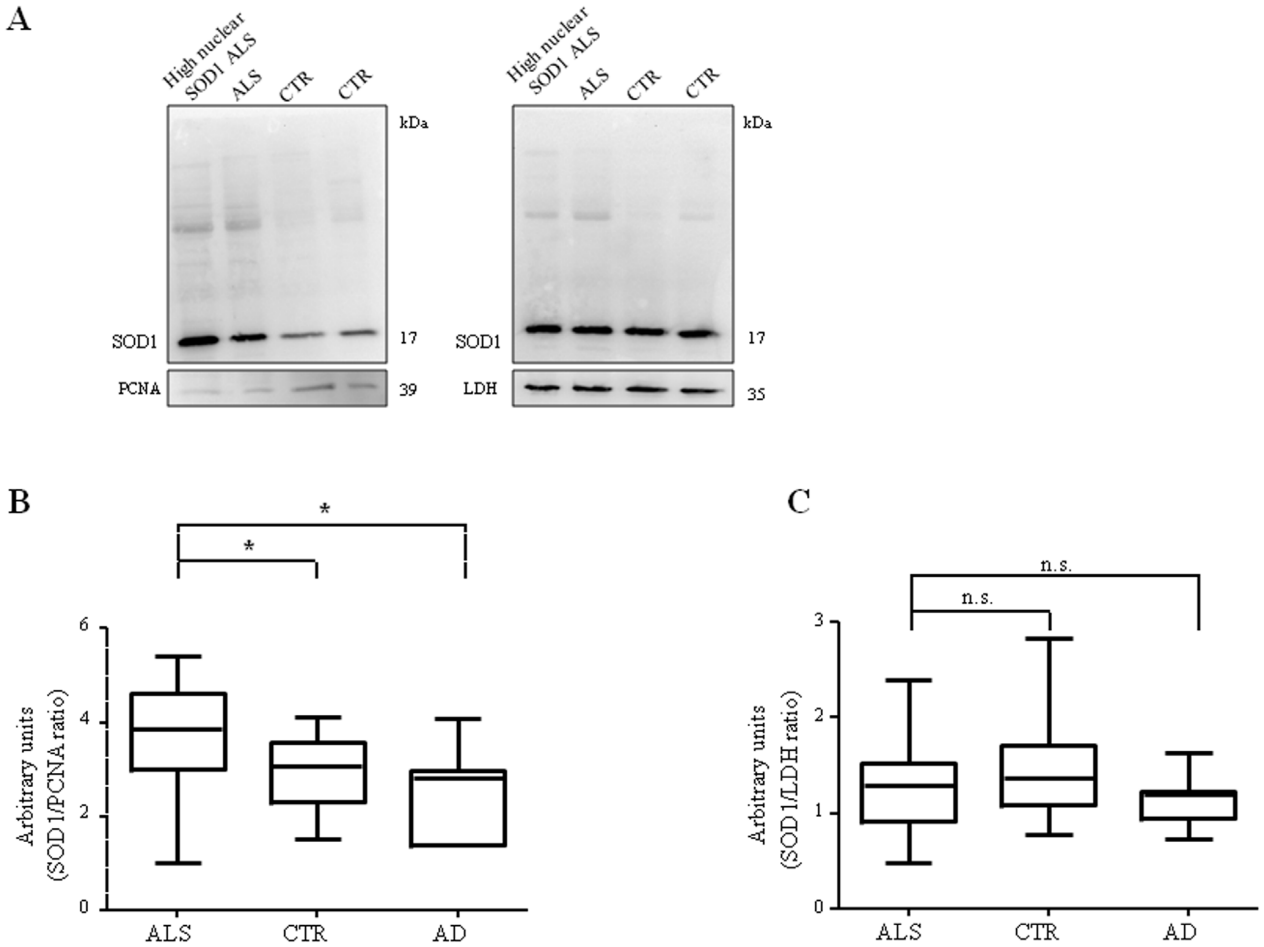
SOD1 expression in nuclear and cytoplasmic soluble fractions of PBMCs from ALS patients, CTR and AD subjects. Representative immunoblotting of SOD1 expression in nuclear (A, left) and cytoplasmic (A, right) fractions from two ALS patients and two controls. Stripped membranes were reincubated with antibody against normalizing nuclear PCNA and cytoplasmic LDH, respectively. Densitometric analysis of SOD1 in nuclei (B) and cytoplasms (C) from ALS, CTR and AD showing SOD1 levels in both the compartments. *p<0.05; n.s., not significant.

SOD1 localization and expression in different cellular compartments was investigated by nuclear and cytoplasmic fractionation of PBMCs from ALS, CTR and AD. Western blot analysis performed on soluble fractions confirmed that SOD1 level is significantly increased (*p*<0.05) in the nuclear compartment of ALS patients (Figures 2A left panel and 2B), compared to CTR and AD. Although nuclear SOD1 was differently expressed between ALS and controls, protein levels were similar in soluble cytoplasmic fractions from ALS patients, neurological and healthy controls (Figures 2A right panel and C). Membranes were also tested to exclude a cross-contamination between the nuclear and cytoplasmic fractions (Figure S3).

### Insoluble SOD1 expression in PBMCs

Confocal images revealed the presence of perinuclear aggregates of the protein in the cytoplasm of PBMCs in a group of ALS patients (Figures 1a, b, c). On the contrary, western blot analysis did not show any difference of SOD1 expression in the soluble cytoplasmic compartment. To test the hypothesis that aggregated forms of SOD1 could be precipitated in the insoluble fraction, we assessed its presence in this compartment by western blot analysis. Experiments were carried out only on SALS and CTR samples as the confocal images have not revealed SOD1 aggregates in PBMCs from AD.

**Figure 3:**
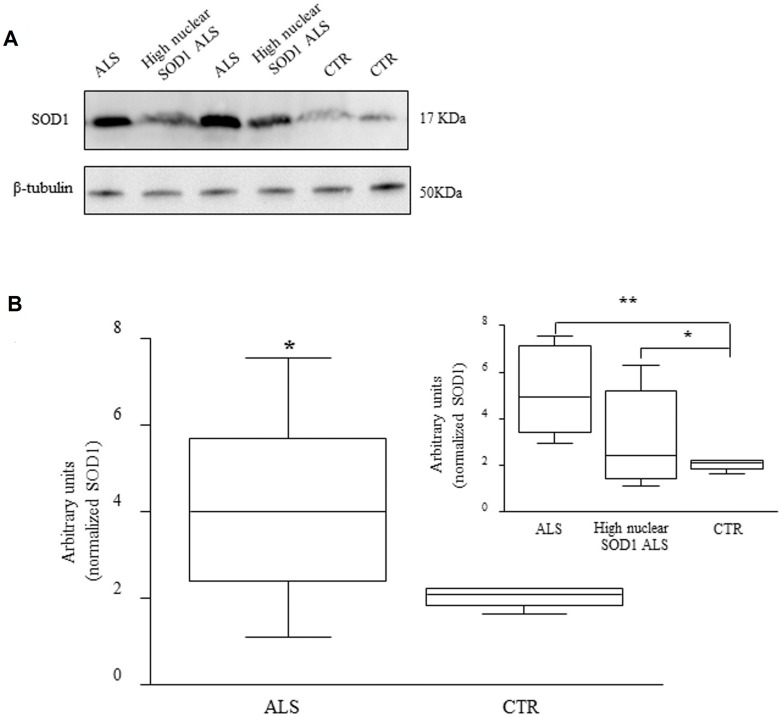
SOD1 expression in the insoluble fraction of PBMCs from ALS patients and CTR. Representative immunoblotting of the insoluble fraction from four ALS patients and two CTR. Stripped membranes were re-incubated with antibody against normalizing protein, β-tubulin (A). Patients show a significantly higher SOD1 level compared to controls (*p<0.05) (B).

Results showed that the amount of SOD1 in the insoluble fraction from ALS patients was statistically higher compared to controls (*p*<0.05). We observed that in some patients SOD1 protein was between 1.5–4 times more expressed than in controls (Figures 3A and B). In agreement with the confocal microscopy results, patients showing the presence of perinuclear aggregates had higher amount of SOD1 protein in the insoluble fraction compared to both controls (*p*<0.01) and patients with higher soluble SOD1 nuclear expression (Figure 3B, insert).

### SOD1 expression in single cells by flow cytometry

**Figure 4:**
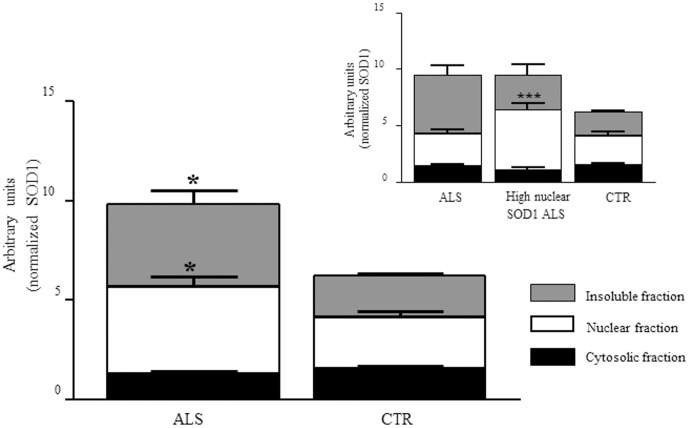
SOD1 expression in three different sub-cellular compartments of PBMCs from ALS patients and CTR. Patients and controls show differences in protein expression profile in nuclear and insoluble fractions (*p<0.05). No differences are reported in soluble cytoplasmic fractions. ALS sub-grouping clearly shows significant differences in SOD1 levels in the cellular compartments (up right panel). Although an overall SOD1 increase is evident in PBMCs from ALS patients, the group with higher nuclear SOD1 has similar level in the other two compartments when compared to CTR.

**Figure 5:**
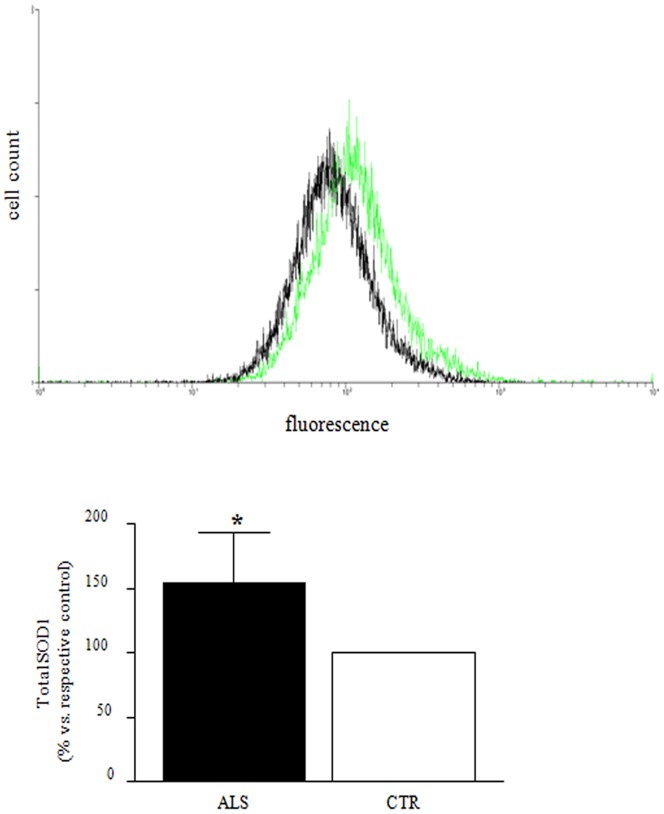
SOD1 expression in isolated PBMCs from ALS patients and CTR using flow cytometry. Curves show SOD1 fluorescence plots in one representative ALS patient (green) and the matched CTR (black) (A). Fluorescence values are normalized to cell count for both ALS and CTR subjects. The shift of the curve on the X axis, expressed in log scale, underlines a difference in SOD1 fluorescence intensity between the two groups, with an overall increased significant signal for ALS compared with CTR (B). *p<0.01.

Immunofluorescence analysis by confocal microscopy showed a higher SOD1 signal in PBMCs from ALS patients. This observation was also proved by western blot experiments considering the three cellular fractions. In figure 4 it is evident that a higher amount of SOD1 is expressed in ALS subjects, also splitting the patients with high nuclear SOD1 from those with normal nuclear expression (Figures 4, insert). To further confirm this overall increase of SOD1, flow cytometry technique was performed in intact PBMCs to evaluate the whole SOD1 protein expression avoiding any precipitation in the insoluble fraction and protein loss that can eventually happen during the recovering process. In these experiments we found a significantly higher fluorescence signal in cells from ALS patients compared to control cells (Figure 5, *p*<0.05) confirming the findings reported above.

### SOD1 expression and correlation with clinical characteristics

The relationship between SOD1 expression in soluble and insoluble fractions and some clinical parameters (i.e. ALSFRS-R score, site of onset, and disease duration) was analyzed. No statistically significant correlations between ALSFRS-R score or site of onset and the nuclear/cytoplasmic SOD1 were evidenced. However, patients with high nuclear SOD1 displayed longer disease duration (in months, mean ± SE: 42.44±14.86 and 34.89±9.98, respectively) (Figure S4).

## Discussion

In a previous paper we described an altered level of SOD1 protein in leukocytes from sporadic ALS patients [26] where a lower SOD1 expression was found in soluble fraction using low-strength detergent buffers. Extending the research to the study of SOD1 mRNA we found an over-expression not only in patients' PBMCs but also specifically in brain affected areas [25]. Immunohistochemistry of affected spinal cord tissue on the same subjects unraveled the presence of increased total SOD1 protein expression in SALS patients compared to CTR individuals [25]. This discrepancy led us to hypothesize that the “missing” protein could be re-localized in different sub-cellular fractions. Specifically, either re-localization in the nucleus or aggregation/precipitation in the insoluble fraction could be supposed. Indeed, SOD1 amount recovered from cell lysates is mostly dependent on buffer formulation that in our previous studies was not strong enough to collect the whole nuclear compartment or the proteins trapped in cytoplasmic inclusions [25]. In the present study we tested both the hypotheses using immunofluorescence and immunoblotting techniques in PBMCs from ALS patients, healthy subjects and neurological controls. Using subcellular fractionation and insoluble protein extraction methods we have been able to investigate SOD1 levels in soluble nuclear and cytoplasmic fractions as well as in insoluble fractions.

Confocal microscopy showed an increased intensity of SOD1 signal in the nuclear compartment in a subset of SALS patients while perinuclear SOD1 aggregates were found in the remaining ALS subjects. Immunoblotting in nuclear soluble fractions confirmed the increased SOD1 levels in accordance with the immunofluorescence analysis. Indeed, SOD1 amount in cytoplasmic soluble fractions showed no difference between ALS patients and controls confirming our previous data on nervous tissue [25]. Western blot analysis of insoluble fractions indicated a higher presence of detergent-resistant SOD1 in those SALS patients characterized by SOD1 perinuclear positive aggregates detected by confocal microscopy. Contrariwise, patients with higher soluble nuclear SOD1 amount displayed insoluble cytoplasmic SOD1 level more similar to controls. This suggests a possible relationship between the presence of protein aggregates and SOD1 enrichment in the insoluble fraction. On the other side, its higher expression profile inside the nucleus is likely related to its higher solubility. In recent works [20], [21], using specific antibodies against misfolded SOD1 protein, some authors found two different staining patterns of SOD1 in motor neurons of sporadic patients: cells with intranuclear SOD1 had fewer aggregates in cytoplasm, while high misfolded cytoplasmic SOD1, located in the perinuclear region, was associated with no nuclear staining. In this work, as we did not use a specific anti-misfolded SOD1 antibody, we cannot state that SOD1 protein is also misfolded. However, from the findings showed here and in a previous paper we have evidence that the commercial antibody used in the current study is able to detect both normally folded and mutant/misfolded SOD1 [36].

In another paper [19], a conformational specific antibody recognizing both oxidized and mut-SOD1 suggested the existence of a common epitope and demonstrated that motor neurons of a subgroup of SALS patients were immunoreactive to this antibody. We recently described the presence of hyper-oxidized aggregated SOD1 in lymphoblasts from sporadic ALS patients [36], suggesting a possible mut-SOD1-like pathogenic mechanism [23] for the oxidized WT-SOD1 protein in peripheral SALS cells.

These works added further evidence to the overall shared hypothesis that conformational anomalies and/or post-translational modifications of WT-SOD1 contribute to ALS pathogenesis as inherited SOD1 mutations do. As already discussed by Forsberg and coworkers, we do not know whether increased nuclear SOD1 represents a physiological or pathological feature in the cellular pathways. Surely, the overall increased SOD1 protein level could be an attempt to protect PBMCs from damages induced by oxidative stress as higher levels of reactive oxygen species were found in PBMCs from ALS patients compare to controls (unpublished data).

Although clear evidence of a nuclear function of SOD1 protein has not been ascertained yet, a possible role at transcriptional level of SOD1 has been recently suggested [40], [41], disclosing others possible activities in addition to its scavenging ability. In a tumor cell line it has been proved that SOD1 counteracts stressful conditions by favoring the binding of transcriptional factors to DNA and inducing the synthesis of proteins with a protective role [40]. Moreover, nuclear SOD1 was essential for viability in conditional SOD1 knockout cells from chicken DT40 and SOD1 depletion increased sister chromatid exchange frequency and the number of apurinic/apyrimidinic sites, suggesting nuclear SOD1 function as a guardian of the genome [41]. Nuclear localization of SOD1 was also important to reduce DNA lesions caused by an increase of the superoxide anion [41]. Using G93A-SOD1 transgenic mice, Sau and coworkers demonstrated that WT-SOD1 was present in cytoplasm and nuclei of motor neurons, whereas G93A SOD1 was mainly cytoplasmic [24]. Similar data have been obtained using NSC34 cells where G93A-SOD1 exclusion from the nucleus produced higher DNA damage compared with those expressing WT-SOD1 [24]. As suggested for mut-SOD1, the shortage of protein at nuclear level could be ascribed to SOD1 entrapment in cytoplasmic aggregates. Consequently, it is reasonable to assume that some non-native conformations of SOD1 cause its precipitation in cytoplasmic inclusions thus limiting the physiological activity of the properly folded SOD1 in the nuclear compartment. The capacity to counteract the progressive aggregation of misfolded SOD1 could be more or less efficient depending on the individual ability to respond to toxic insults and thus generating the clinical differences observed in ALS course.

An association between short disease duration and high aggregation propensity has been proved for the mutant SOD1 [42]. In agreement with these findings, it is important to remark that in our study we found longer disease duration in the group with higher amount of soluble SOD1 within the nucleus, suggesting a possible protective role of the protein in this compartment. However, these data need to be confirmed in a larger group of patients and in a longer follow-up study. No significant correlation between increased SOD1 nuclear localization or its presence in the insoluble fraction and site of disease onset was found in our study. This differs from what we previously found in lymphoblasts [36] where the presence of a misfolded and aggregated SOD1 correlated with bulbar disease onset. This difference could be explained by the diverse cellular model employed in the two studies. Indeed, in lymphoblasts an increasing progressive accumulation of the damage overtime can be observed, while PMBCs are continuously replaced, especially if damaged.

In short, by this work an increased expression of total SOD1 protein seems evident in PBMCs of SALS patients. This data are in line with mRNA and protein expression results previously found [25], [43]. In addition, the increased expression of SOD1 observed in PBMCs represents a novelty as fractionation of lysates from PBMCs have not been performed so far. The augmented SOD1 protein could have two fates in the physiopathological mechanisms of ALS: to remain soluble and able to exert its function(s) in the nucleus or to aggregate in the cytoplasm. Both literature evidence and the observations here reported lead us to suppose that increased accumulation of nuclear soluble SOD1 represents a potential protective cellular reaction. In the subgroup of patients with augmented nuclear SOD1, the disease could likely be caused by other independent primary pathogenic events (e.g. aberrant localization of FUS and/or TDP-43) [44]. Otherwise, the increased insolubility of the protein could impair/prevent both the nuclear localization and its physiological functions through its accumulation in cytoplasmic aggregates that could involve several fundamental ALS proteins with aberrant localization [45]. Nevertheless, the hypothesis of enhanced accumulation of soluble SOD1 in the nucleus as a possible “defensive” mechanism deserves further in-depth investigation since it may lead to novel therapeutic approaches based on the pharmacological targeting of SOD1 sub-cellular distribution as well as on the modulation of its solubility.

## Supporting Information

Figure S1 **Distribution of normalized values of nuclear SOD1 in ALS and controls.** Histograms display the bimodal distribution of the normalized values corresponding to the nuclear SOD1 in ALS patients whereas a bell-shaped distribution was obtained for the controls.
(TIF)

Figure S2 **High magnification of confocal images of SOD1 distribution in PBMCs from ALS and CTR subjects.** Immunofluorescence images reveal SOD1 cytoplasmic aggregates in PBMCs from SALS patients with lower SOD1 nuclear distribution while in other patients we observed cells with higher SOD1 signal in the nuclear compartment.
(TIF)

Figure S3 **Representative western blots showing the absence of cross-contamination between the two subcellular fractions.** Nuclear fractions were tested for the presence of the cytoplasmic protein LDH while the presence of cytoplasmic contamination in nuclear fractions was tested using the nuclear protein PCNA. The level of cross-contamination between the two subcellular fractions is very low; hence, no significant cross-contamination occurs between the two extracts.
(TIF)

Figure S4 **Correlation between SOD1 nuclear expression and disease duration.** **A** linear regression analysis was conducted relating normalized values of nuclear SOD1 and disease duration. A positive significant correlation (r = .588; p = .008) was found between the two parameters.
(TIF)
