# Supplementary figures and images for: Altered Intracellular Localization of SOD1 in Leukocytes from Patients with Sporadic Amyotrophic Lateral Sclerosis
Source: PLoS One. 2013 Oct 14;8(10):e75916. doi: 10.1371/journal.pone.0075916 (PMC3796534; doi:10.1371/journal.pone.0075916)

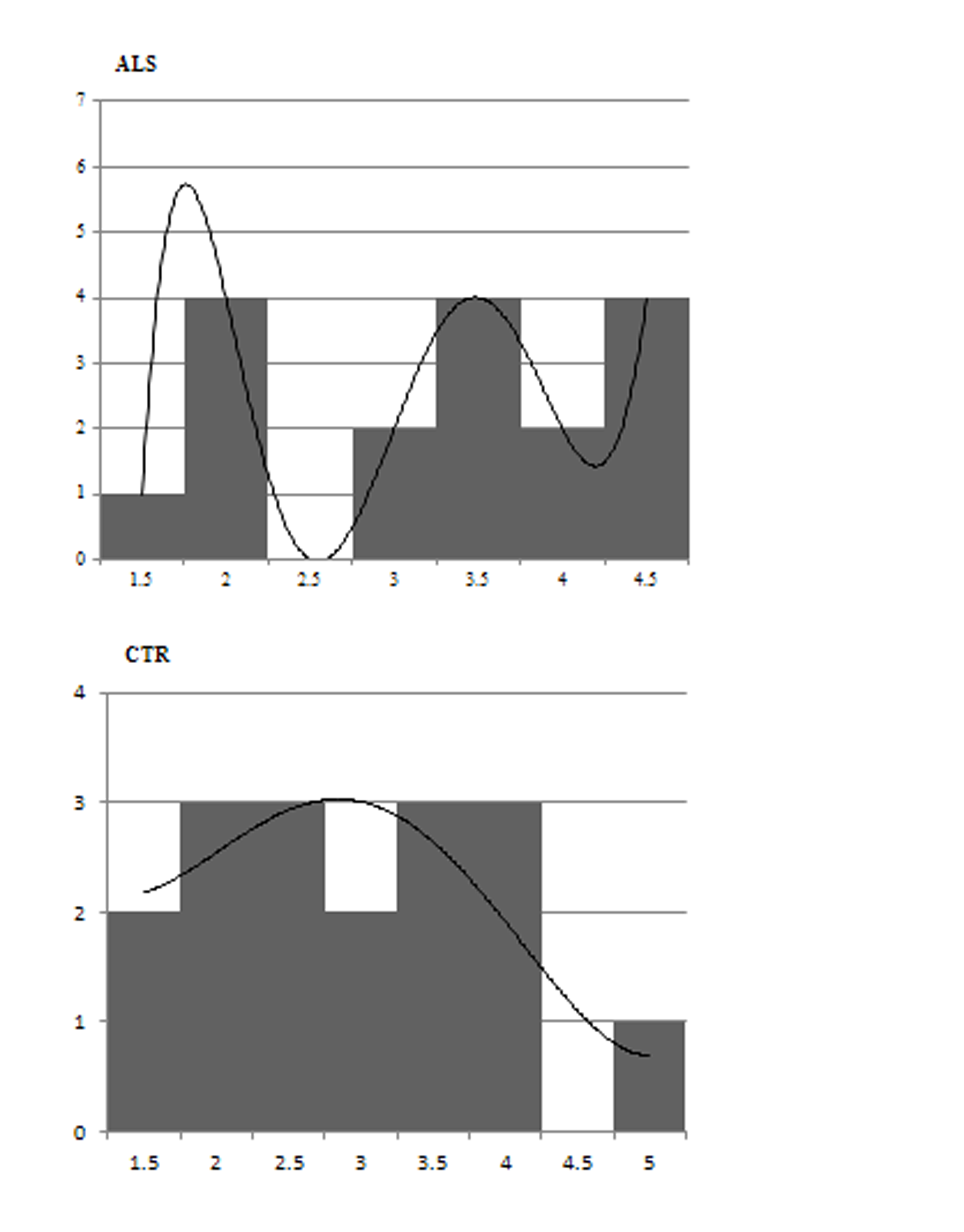

Supplement: Figure S1 — Distribution of normalized values of nuclear SOD1 in ALS and controls. Histograms display the bimodal distribution of the normalized values corresponding to the nuclear SOD1 in ALS patients whereas a bell-shaped distribution was obtained for the controls. (TIF) [file pone.0075916.s001.tif]

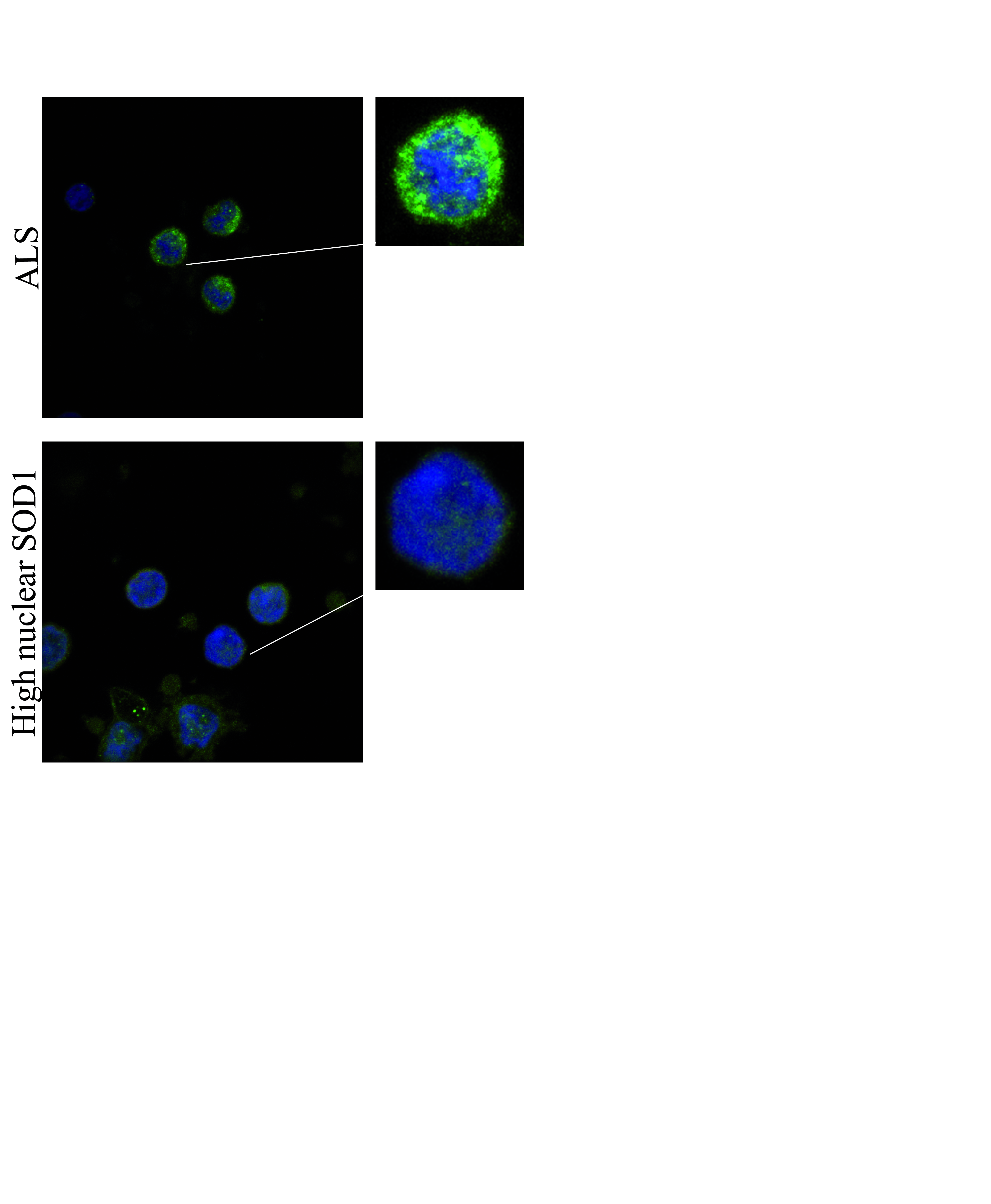

Supplement: Figure S2 — High magnification of confocal images of SOD1 distribution in PBMCs from ALS and CTR subjects. Immunofluorescence images reveal SOD1 cytoplasmic aggregates in PBMCs from SALS patients with lower SOD1 nuclear distribution while in other patients we observed cells with higher SOD1 signal in the nuclear compartment. (TIF) [file pone.0075916.s002.tif]

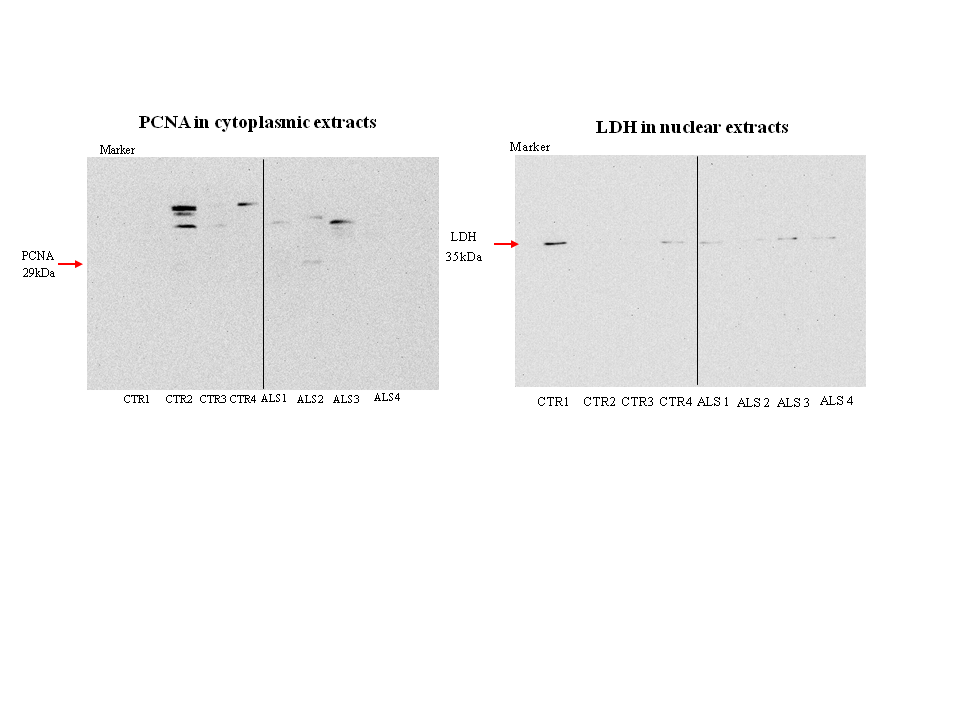

Supplement: Figure S3 — Representative western blots showing the absence of cross-contamination between the two subcellular fractions. Nuclear fractions were tested for the presence of the cytoplasmic protein LDH while the presence of cytoplasmic contamination in nuclear fractions was tested using the nuclear protein PCNA. The level of cross-contamination between the two subcellular fractions is very low; hence, no significant cross-contamination occurs between the two extracts. (TIF) [file pone.0075916.s003.tif]

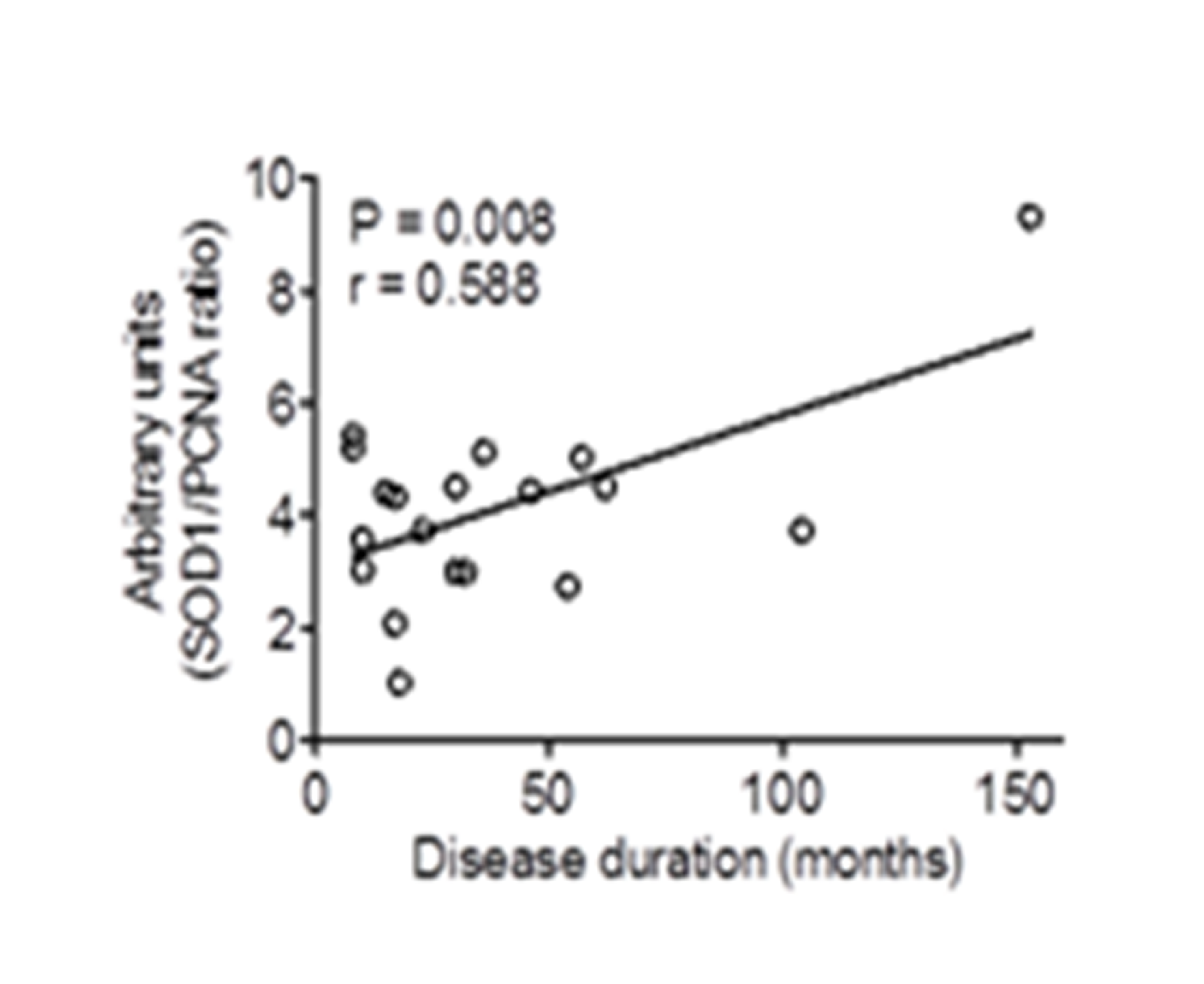

Supplement: Figure S4 — Correlation between SOD1 nuclear expression and disease duration. A linear regression analysis was conducted relating normalized values of nuclear SOD1 and disease duration. A positive significant correlation (r = .588; p = .008) was found between the two parameters. (TIF) [file pone.0075916.s004.tif]
